# Spin injection in n-type resonant tunneling diodes

**DOI:** 10.1186/1556-276X-7-592

**Published:** 2012-10-25

**Authors:** Vanessa Orsi Gordo, Leonilson KS Herval, Helder VA Galeti, Yara Galvão Gobato, Maria JSP Brasil, Gilmar E Marques, Mohamed Henini, Robert J Airey

**Affiliations:** 1 Physics Department, Federal University of São Carlos, São Carlos 13.565-905, Brazil; 2 Gleb Wataghin Physics Institute, UNICAMP, Campinas, 13083-970, Brazil; 3 School of Physics and Astronomy, University of Nottingham, Nottingham, NG7 2RD, UK; 4 Department of Electronic and Electrical Engineering, University of Sheffield, Sheffield, S10 2TN, UK

**Keywords:** Spintronics, nanostructure, resonant tunneling diode, photoluminescence

## Abstract

We have studied the polarized resolved photoluminescence of n-type GaAs/AlAs/GaAlAs resonant tunneling diodes under magnetic field parallel to the tunnel current. Under resonant tunneling conditions, we have observed two emission lines attributed to neutral (X) and negatively charged excitons (X^−^). We have observed a voltage-controlled circular polarization degree from the quantum well emission for both lines, with values up to −88% at 15 T at low voltages which are ascribed to an efficient spin injection from the 2D gases formed at the accumulation layers.

## Background

In the last years, it has been an increasing interest in the manipulation of spin degrees of freedom in semiconductor devices. Particularly, some attention was focused on non-magnetic resonant tunneling diodes (RTDs) because the spin polarization of carriers in the structure can be voltage-controlled which is very useful for device applications [1-11]. However, the voltage dependence of the polarization degree is not well understood and it seems to depend on various contributions such as filling factors, spin injection from the two dimensional (2D) gases formed in the accumulation layers next to the barriers, and charge accumulation in the quantum well (QW). In this paper, we have studied spin effects in a non-magnetic n-type GaAs/AlGaAs resonant tunneling diode (RTD). The spin polarization of carriers was studied by analyzing the current-voltage characteristics curve (*I-V*) and the right (σ^+^) and left (σ^−^) circularly polarized photoluminescence (PL) from the contact layers and the QW as function of applied voltage under magnetic fields up to 15 T. We have investigated the polarization degree of both the QW and 2D gases emissions. Under applied voltage and light excitation, electrons and photo-generated holes tunnel through the double-barrier structure creating a 2D electron and a hole gas at the accumulation layers next to the barriers. These 2D gases can inject spin-polarized carriers into the QW under applied voltage resulting in high polarization degree values. This injection seems to be very efficient at low voltages. However, under higher voltages, other effects probably contribute to the spin polarization of carriers in the QW, including charged exciton or trion formation. In previous works, structures with smaller QW widths were used, therefore emission from trions were not resolved in the PL spectra [4-8]. In this work, we have studied a device with a larger QW width which has revealed the formation of trions in the QW. Our results show that the QW circular polarization degree for charged and neutral excitons is voltage dependent with relatively higher values, up to −88% at 15 T for low bias voltages. This result cannot be attributed solely to a simple thermal occupation effect and is mainly attributed to the injection of polarized carriers from the contacts. Under higher voltages, the QW circular polarization is reduced, indicating additional contribution for the spin polarization in the QW such as the increasing of the density of carriers and the formation of trions in the QW.

## Methods

PL measurements were performed by using a Si charge-coupled device to an Andor Shamrock SR-500i spectrometer (Andor Technology, Belfast, UK). The right σ^+^ and left σ^−^circularly polarized PL were selected by using a quarter wave retarder and a polarizer. A linearly polarized 532-nm continuous wave laser was used for optical excitation. As a consequence, the photo-generated carriers do not present a defined spin polarization. Optical and transport measurements were performed at the temperature of 4 K under magnetic fields parallel to the tunnel current. Our n-type RTD structure was grown by molecular beam epitaxy on an n + (001) GaAs substrate. It consists of 0.6 μm n-GaAs (10^18^ cm^*−*3^), 806 Å n-GaAs (10^17^ cm^*−*3^), 509 Å n-GaAs (10^16^ cm^*−*3^), 209 Å undoped GaAs spacer, 57 Å Al _0*.*4_ Ga_0*.*6_ As barrier, 90 Å GaAs QW, 57 Å Al _0*.*4_ Ga _0*.*6_ As barrier, 209 Å GaAs spacer, 509 Å n-GaAs (10^16^ cm^*−*3^), 806 Å n-GaAs (10^17^ cm^*−*3^), and 2.0 μm n-GaAs (10^18^ cm^*−*3^). The devices were processed in circular mesas of about 200 μm diameter with annular AuGe contacts allowing optical measurements.

## Results and discussion

**Figure 1:**
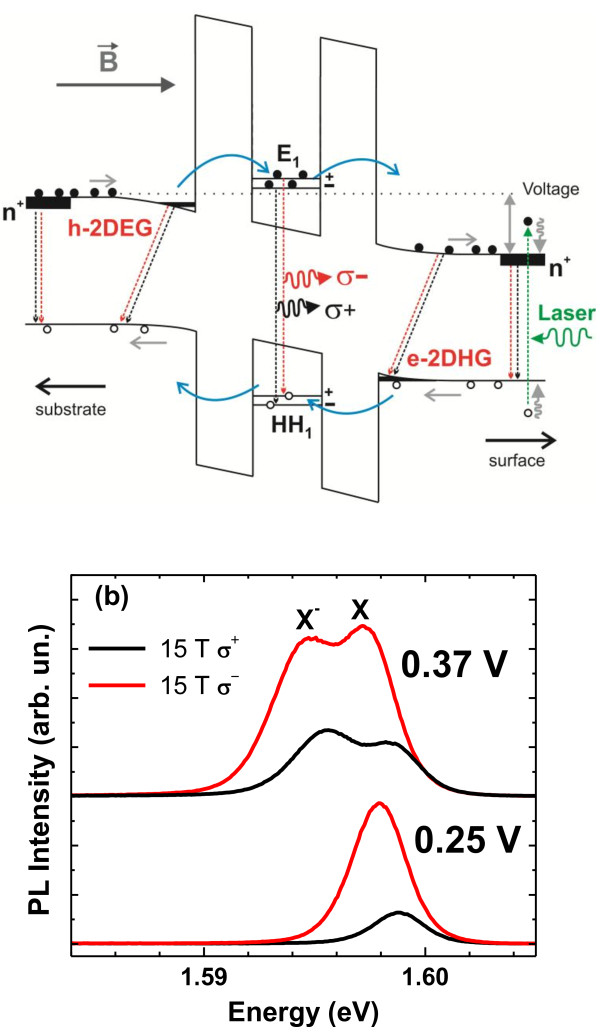
**Schematic band diagram of the n-type RTD and typical PL emission from the QW. **(**a**) Schematic band diagram of the n-type RTD under forward bias, light excitation, and magnetic field parallel to the tunnel current. (**b**) Typical PL emission from the QW for different voltages and 15T.

A schematic band diagram of our device under forward bias voltage, magnetic field, and light excitation is shown in Figure 1a. Under this condition, carriers can tunnel and recombine at different layers of the structure. The confined levels in the QW and the contact layers split into spin-up and spin-down Zeeman states, and the optical recombination can occur with the defined selection rules giving information about the spin polarization of the carriers in the structure. The PL spectra include the emission from the QW and the contact layers, and both are voltage dependent. It is well known that the relative concentrations of electrons and holes in the QW of RTD structures can be controlled by external parameters [12-17]. By varying the applied bias and the laser intensity, it is possible to change significantly the concentration of electrons and photo-generated holes tunneling through the barriers of our structure. As a consequence, the QW PL emission can comprise neutral, positively charged (X^+^), and negatively charged (X^−^) excitons, also known as trions [12-17]. In n-type RTDs, the X^+^ is typically observed for voltages near photo-generated hole resonances (hole-rich region), and the X^−^ emission is observed for voltages around electron resonances (electron-rich region) [15]. The contact layer emission comprises additional transitions that originate from indirect transitions from the two-dimensional electron (2DEG) and hole gases (2DHG) created next to the barriers. We remark that those 2D gases have voltage-controlled g-factors and carrier densities. Under magnetic fields, the 2D gases are usually strongly spin-polarized [18-21] and can contribute to the polarization of the carriers in the QW by injecting preferentially polarized carriers at different applied biases. The PL spectra are very sensitive to the variation of charge density and therefore will be voltage dependent.

Typically polarized resolved QW PL spectra at 15 T from our device are shown in Figure 1b. We observed that QW PL presents two peaks. These peaks are solely observed around the electron resonance. As shown in Figure 1b, the voltage dependence of these peaks is consistent with the attribution of the negatively charged exciton or X^−^ (the lower energy peak) and the neutral exciton (X) (the higher energy peak). For our experimental conditions, the X^−^ ground state should be the singlet state which consists of one hole and two electrons with antiparallel spins.

**Figure 2:**
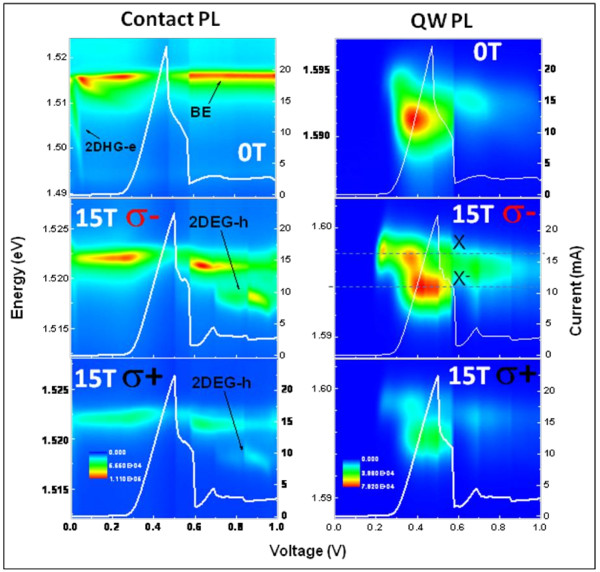
***I-V *characteristics curve and color-coded maps of polarization-resolved PL intensities. ***I-V* characteristics curve and color-coded maps of polarization-resolved PL intensities as a function of voltage for contact layers (left image) and QW under *B* = 15 T (right image).

The current-voltage characteristics *I-V* curve and the color-coded maps of the polarized resolved PL emission as a function of bias voltage for the contact layers, and the QW is shown in Figure 2 for *B* = 0 and 15 T. *I-V* curves present a dominant peak associated to the electron-resonant tunneling condition. Under magnetic field, the additional peaks are revealed after the major resonance and are attributed to the inelastic scattering-assisted resonant tunneling [22]. The optical emission from the GaAs contact layers includes several bands represented on Figure 1: the bulk-exciton transition from the undoped GaAs spacer layers, the recombination between photo-generated holes and donor-related electrons from the n-doped GaAs layers, and the indirect recombination between free holes (electrons) and confined electrons (holes) localized at the 2DEG (or 2DHG) formed at the accumulation layer next to the barriers (labeled 2DEG-h and 2DHG-e emissions). We point out that the 2DHG-e emission is observed both without and under applied magnetic fields. However, this emission is only observed for low voltages, and as for the larger voltages, the relatively small reservoir of photo-created holes (2DHG) accumulated at the top barrier interface must be mainly depleted. In contrast, the 2DEG-h emission is only observed under high magnetic fields and higher voltages. Its intensity (Figure 2) presents an abrupt increase at 0.85 V which is consistent with the increasing electron density accumulated at the 2DEG just after the resonant tunneling condition. For larger voltages, the 2DEG-h emission tends to vanish, which may be associated to a reduced efficiency on the localization of holes around the 2DEG due to the significantly large electric field or to reaching a critical density of electrons at the 2DEG. Figure 2 shows that the QW PL intensity increases with increasing applied voltages during the resonant condition and decreases after the resonant tunneling condition. Particularly, the QW PL intensity presents a good correlation with the *I-V* characteristics curve due to the voltage-controlled electron/hole carrier density at the QW.

**Figure 3:**
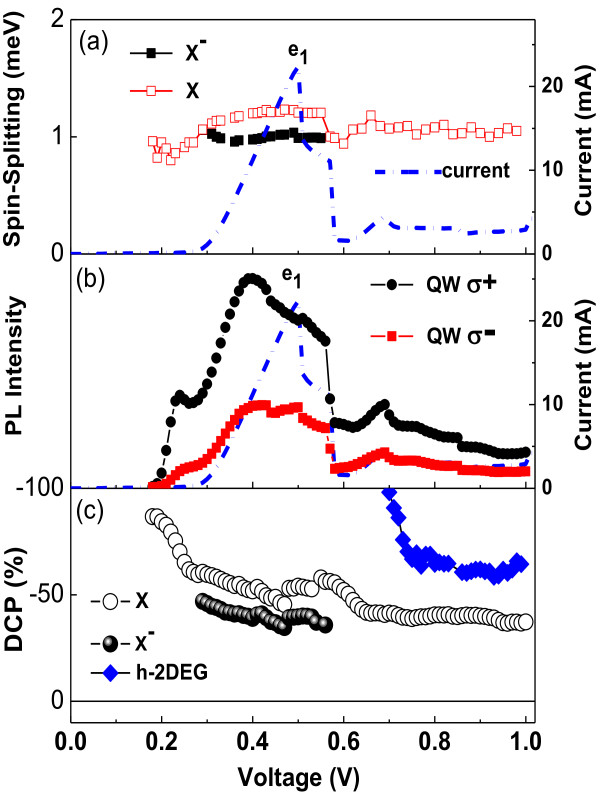
***I-V *characteristics curve and voltage dependence of polarization resolved photoluminescence. **(**a**) *I-V* characteristics curve and voltage dependence of X and X-spin splitting of the PL spectra, (**b**) voltage dependence of the total integrated QW PL intensity and (**c**) circular polarization degree of excitons and trions in the QW and of the 2DEG-h emission.

Figure 3a presents the *I-V* characteristics curve and the voltage dependence of the spin-splitting energy for the X and X^−^ emission lines. The X^−^ results are presented only for the range where this emission is observed around the electron resonance. We observed that the spin splitting of the neutral exciton presents some correlation with the *I-V* curve. This behavior was previously observed and is associated to a variation the effective electric field in the QW region [4]. Figure 3b presents the voltage dependence of the total integrated QW PL intensity. As discussed before, the total integrated QW PL intensity presents a much clear correlation with the *I-V* curve for both σ^+^ and σ^−^ emissions. Particularly, the peak of PL intensity is observed around 0.2 V, which is associated to a photo-generated hole resonant tunneling. This hole resonance is only observed in the *I-V* curve under higher laser intensities (not shown). After the electron resonance, we observed several peaks attributed to the satellites peaks due to inelastic scattering-assisted resonant tunneling.

The circular polarization degree was calculated by the following equation:

$$P=\left(I^{\sigma +}-I^{\sigma -}\right)/\left(I^{\sigma +}+I^{\sigma -}\right)$$

where *I*^σ+^ (*I*^σ−^) are the integrated intensity of the right (left) circular polarization. Figure 3c presents the circular polarization degree obtained from the QW and 2DEG-h emissions under 15 T and 4 K. The polarization degree from 2DHG-e optical emission is not presented because this emission becomes too weak under high magnetic field. We have observed that the polarization degree from free exciton and negatively charged exciton in the QW are voltage dependent. Particularly, we have observed that the X^−^ polarization degree is slightly lower than the X polarization degree. If we suppose that our system is an isolated QW under thermal equilibrium, we should expect some difference in the polarization degree of the PL emission from trions and excitons due to their different g-factors and lifetimes [23-25]. In our case, the situation is even more complex, as our system has some particularities if we compare to an isolated QW. The generation of carriers at the RTD QW is dominated by tunneling of carriers from the accumulation layers. Those carriers can be either photo-created at the contact layers or originate from doping. Therefore, the generation rates of carriers with distinct spin polarizations at the RTD QW should depend on the g-factor at the accumulation layers and the tunneling efficiencies for carriers with distinct spin polarizations. Furthermore, the thermal equilibrium is no longer a good approximation, i.e., we should use modified lifetimes that depend not only on the recombination time, but also on the tunneling times in a complex form.

Consequently, a simple evaluation of the polarization degree considering the thermal equilibrium and conventional parameters from regular GaAs QWs is not realistic for our structure. It is important to point out that clearly the voltage dependence of the QW polarization degree does not follow the voltage dependence of the spin-splitting energy from this emission (Figure 3a). Therefore, it cannot be attributed to a simple thermal occupation effect of the QW excitonic states. Figure 3b shows that the polarization degree of 2DEG-h emission is also voltage dependent. In general, the increase of the applied voltage on the RTD results in strong variations of the carrier densities at the accumulation layers and, therefore, in the changes of the filling factors of 2D gases in the structure. The 2DEG-h polarization degree is higher than the QW polarization degree which indicates some spin polarization loss on the tunneling processes probably due to the efficient scattering processes in this voltage region. However, a quantitative analysis of the QW polarization must also consider additional effects, including the thermal occupation of the QW levels, the trion formation, and the loss/gain of spin polarization during the tunneling processes.

## Conclusions

In conclusion, we have observed that the polarization degree from neutral and charged excitons in the QW and from the 2D gases emissions formed at the accumulation layers of n-type RTDs are voltage dependent. Under applied bias and magnetic field, the resonant tunneling diode creates a highly spin-polarized two-dimensional gas which seems to act as a spin-polarized source of injected carriers to the structure. The spin injection to the QW seems to be especially efficient under low voltages (before electron resonance) when we observe a clear discrepancy between the relatively small spin-splitting energy and a rather large circular polarization degree. Under higher voltages, the QW polarization may depend on other additional effects, including trion formation and the loss/gain of spin polarization during the tunneling processes in and out of the QW.

## Competing interests

The authors declare that they have no competing interests.

## Authors' contributions

VOG carried out the PL, transported the measurements, and prepared the figures. LKSH carried out the PL and transport measurements. HVAG prepared the figures and participated in the analyses of the data.YGG conceived the study, interpreted the data, and wrote the paper. GEM drafted the manuscript. MJSPB participated in the interpretation of the results and drafted the manuscript. MH is responsible for the growth of the RTD and RJA for its processing. All authors read and approved the final manuscript.

## Authors’ information

VOG and LKSH are PhD students of the Physics Department at the Federal University of São Carlos, Brazil. HVAG is a FAPESP post-doctoral fellow at the Federal University of São Carlos, Brazil.YGG and GEM are professors at the Federal University of São Carlos, Brazil. MJSPB is a professor at UNICAMP, Brazil. MH is a professor of Applied Physics at the University Nottingham, UK. RJA is a research associate at the University of Sheffield.
